# Synthesis and Mechanism of Metal-Mediated Polymerization of Phenolic Resins

**DOI:** 10.3390/polym8050159

**Published:** 2016-04-26

**Authors:** Zhao Yi, Jizhi Zhang, Shifeng Zhang, Qiang Gao, Jianzhang Li, Wei Zhang

**Affiliations:** 1 Ministry of Education (MOE) Key Laboratory of Wooden Material Science and Application, Beijing Key Laboratory of Wood Science and Engineering, School of Materials Science and Technology, Beijing Forestry University, Beijing 100083, China; wangw1007@bjfu.edu.cn (Z.Y.); shifeng.zhang@bjfu.edu.cn (S.Z.); gaoqiang@bjfu.edu.cn (Q.G.); 2 Key Laboratory for Liquid-Solid Structural Evolution and Processing of Materials (MOE), School of Materials Science and Engineering, Shandong University, Jinan 250061, China; zjzvip@sdu.edu.cn

**Keywords:** PF resins, metal catalysts, phenol *ortho* position, synthesis mechanism, ion-polymer

## Abstract

Phenol-formaldehyde (PF) resin is a high performance adhesive, but has not been widely developed due to its slow curing rate and high curing temperature. To accelerate the curing rate and to lower the curing temperature of PF resin, four types of metal-mediated catalysts were employed in the synthesis of PF resin; namely, barium hydroxide (Ba(OH)_2_), sodium carbonate (Na_2_CO_3_), lithium hydroxide (LiOH), and zinc acetate ((CH_3_COO)_2_Zn). The cure-acceleration effects of these catalysts on the properties of PF resins were measured, and the chemical structures of the PF resins accelerated with the catalysts were investigated by using Fourier transform infrared (FT-IR) spectroscopy and quantitative liquid carbon-13 nuclear magnetic resonance (^13^C NMR). The results showed that the accelerated efficiency of these catalysts to PF resin could be ordered in the following sequence: Na_2_CO_3_ > (CH_3_COO)_2_Zn > Ba(OH)_2_ > LiOH. The catalysts (CH_3_COO)_2_Zn and Na_2_CO_3_ increased the reaction activity of the phenol *ortho* position and the condensation reaction of *ortho* methylol. The accelerating mechanism of (CH_3_COO)_2_Zn on PF resin is probably different from that of Na_2_CO_3_, which can be confirmed by the differences in the differential thermogravimetric (DTG) curve and thermogravimetric (TG) data. Compared to the Na_2_CO_3_-accelerated PF resin, the (CH_3_COO)_2_Zn-accelerated PF resin showed different peaks in the DTG curve and higher weight residues. In the synthesis process, the catalyst (CH_3_COO)_2_Zn may form chelating compounds (containing a metal-ligand bond), which can promote the linkage of formaldehyde to the phenolic hydroxyl *ortho* position.

## 1. Introduction

Phenol-formaldehyde (PF) is a high-performance resin that is synthesized by the copolymerization of phenol with formaldehyde. It is widely applied for industrial uses, including adhesives, impregnating resins, and plastics. The excellent properties of PF resin include high mechanical, thermal, and weather stability [1]. However, the lower curing rate and required higher curing temperature compared to other thermosetting adhesives limit the application of PF resins for use in impregnating resins or adhesives [2,3]. Many attempts have been made to accelerate the curing rate or lower the curing temperature, including testing of various catalysts or additives to alter the reaction kinetics, such as carboxylic acid esters [4,5], anhydrides [6], amides [7], carbonate [8], and metallic ions [9]. Additionally, the effects of the condensation condition on the PF resin structure and properties have been well studied by conventional analytical techniques. For example, various mechanisms of PF resin hardening accelerated by catalysts or additives have been reported [10]. Some additives, such as sodium carbonate, act solely to accelerate the curing reaction, but other additives, such as propylene carbonate, both accelerate the reaction and also increase the average functionality of the PF reaction system to allow a tighter final network [10]. The properties of basic catalysts, such as the valence and ionic radius of hydrated cations, affected the mechanisms and kinetics of PF resin condensation and, thus, the composition of the final products [9]. Some studies also reported that an alkaline catalyst promoted the formation of dimethylene ethers in the polymerization reaction, and that *ortho* to *ortho* (*o*,*o*′) ethers were more stable [11]. However, there has been no comprehensive study about the action of catalysts to increase or decrease the ratio of *ortho*/*para* reaction position or analysis of the corresponding physicochemical properties of the accelerated PF resins.

The aromatic ring of phenol has *ortho* and *para* positions capable of reaction with formaldehyde under certain conditions, but the *para* position has higher reactivity than the *ortho* position. The presence of two *ortho* positions and one *para* position in an aromatic ring generally could lead to a PF resin containing mostly *ortho* hydroxymethyl groups [2]. However, in the process of PF resin synthesis, some catalysts could make more formaldehyde or methylol toward phenol *ortho* positions to increase the ratio of *ortho*/*para* substituted positions [12], leading to more reactive functional groups or more unreacted *para* positions at the curing stage, which may shorten the curing time and increase the cross-linking degree of cured PF resin.

Since metal ions can accelerate the curing of PF resins, we tested the ability of barium hydroxide (Ba(OH)_2_), sodium carbonate (Na_2_CO_3_), lithium hydroxide (LiOH), and zinc acetate ((CH_3_COO)_2_Zn) to decrease curing temperature and accelerate the curing rate of PF resins. To elucidate the chemical structure of the cure-accelerated PF resins, we performed quantitative liquid ^13^C NMR to analyze the structural features. Finally, possible synthesis mechanism of metal-mediated polymerization of PF resins was proposed based on the chemical structure analysis and thermogravimetric (DTG) curve.

## 2. Materials and Methods

### 2.1. Materials

Phenol, formaldehyde (37%), Ba(OH)_2_, Na_2_CO_3_, LiOH, and (CH_3_COO)_2_Zn were obtained from Zhong’an Chemical Industries, Beijing, China and were used directly without further purification, and all other chemicals were AR grade and obtained from Beijing Chemical Industries, Beijing, China.

### 2.2. Preparation of PF Resins

The catalyst-accelerated PF resin was synthesized by batch polymerization with phenol and formaldehyde at a molar ratio of 1:2.2, and the additive amount of catalyst was 6% based on the total mass of PF resin. In the first step, phenol was mixed in a flask with one third of the formaldehyde and one third of the catalyst. The mixture was quickly heated to 70 °C, and then the heater was turned off. The temperature of the mixture increased to 90 °C due to the heat produced by polymerization reaction and remained at 93–95 °C for 1 h. In the second step, the remaining formaldehyde and two thirds of the catalyst were added to the flask and the mixture was heated to 90 °C and kept at that temperature for 0.5 h. Finally, the mixture was cooled to 40 °C to yield PF resin. PF resins with different catalysts were synthesized with the same procedure.

### 2.3. Preparation of Plywood

Three-layer plywood (400 mm × 400 mm × 4.8 mm) was prepared with a single poplar veneer in the middle and two poplar veneers on the top and bottom simulating actual industrial parameters. The middle poplar veneer was coated with 125–150 g/m^2^ resin on each side. Four pieces of three-layer plywood for each catalyst-accelerated resin (including control) were hot-pressed under 1.2 MPa at 100, 110, 120, and 130 °C, respectively. The hot-press time was 7 min, including the first one minute and the last one minute to load and unload the pressure, respectively.

### 2.4. Characterization of PF Resins

The solid (non-volatile) content of resol resin was determined in accordance with ASTM standard D4426-01. The viscosity of resin was measured using a Brookfield DV-II viscometer (AMETEK-BROOKFIELD Corporation, Middleboro, MA, USA) using 61# rotor with spinning rate of 100 rpm. Gel time was defined as the time period from the immersion of the test tube into the oil bath (135 °C) to the beginning of the resin gelation (resin forming a string when a glass rod was lifted from the resin).

### 2.5. Characterization of the Plywood

The shear strength was measured as per ASTM D906-98.

### 2.6. FT-IR Analysis of PF Resins

The resins were placed in 0.01 MPa vacuum at 60 °C for 4 h to dry to non-volatility. FT-IR spectra of vacuum-dried PF resins were performed in a Nicolet IS10 instrument (Thermo Fisher Scientific Corporation, ‎Waltham, MA, USA). Each spectrum was recorded with 32 scans in a frequency range of 600–4000 cm^−1^ at a spectral resolution of 4 cm^−1^.

### 2.7. Contact Angle Measurement

The contact angle measurements of the PF resins were performed on the tangential surfaces of wood samples with an optical contact angle apparatus (OCA 20 DataPhysics Instruments GmnH, Filderstadt, Germany). Sessile droplets (3 μL, measured with a microsyringe) of liquid resin were placed on the wood surface. The right and left angles of the drops on the surface were collected at intervals of 0.1 s for a total duration of 60 s, and the average angle was calculated.

### 2.8. Quantitative Liquid ^13^C NMR Measurement

All of the resins were characterized by quantitative ^13^C NMR spectroscopy with a VARIAN INOUR-300 (JEOL Corporation, Tokyo, Japan) spectrometer with a frequency of 75.51 MHz using the inverse-gated decoupling method. All of the spectra were recorded at room temperature with a delay time of 8 s, a 13 h acquisition time and a 15.4 μs pulse width (90°). About 8000 scans were accumulated to obtain spectra for each spectrum. The chemical shifts of each spectrum were accurate to 0.1 ppm and all the resin samples were directly used for ^13^C NMR measurement.

### 2.9. Thermogravimetric Analysis (TG) of Resins

Samples were dried at 120 °C for 2 h to evaporate the moisture and then TG was performed in a nitrogen atmosphere within a temperature range from room temperature to 700 °C, with a heating rate of 10 °C/min.

## 3. Results and Discussion

### 3.1. Performance of the Catalyst-Accelerated PF Resin

Table 1 shows the solid content, viscosity, and gel time of the PF resins. We found that the solid contents were similar for all resins, but the viscosity varied greatly for different catalysts. The viscosity of the Na_2_CO_3_-accelerated resin was 153.00 mPa·s, higher than the viscosity of the other catalyst-accelerated PF resins, especially the control resin with a low viscosity of 25.70 mPa·s. Similarly, the gel-time for the catalyst-accelerated resins varied from 11.83 min for the Na_2_CO_3_-accelerated resin to 20.46 min for the control resin. These results indicated that these catalysts were able to accelerate the synthesis reaction to different extents. The ability of the catalysts to accelerate the reactions could be ranked as Na_2_CO_3_ > (CH_3_COO)_2_Zn > Ba(OH)_2_ > LiOH. Under the same reaction conditions, the catalyst Na_2_CO_3_ can dramatically accelerate the synthesis reaction process, increase the viscosity, and decrease the gel time of PF resin.

### 3.2. Contact Angle of the PF Resins

**Figure 1:**
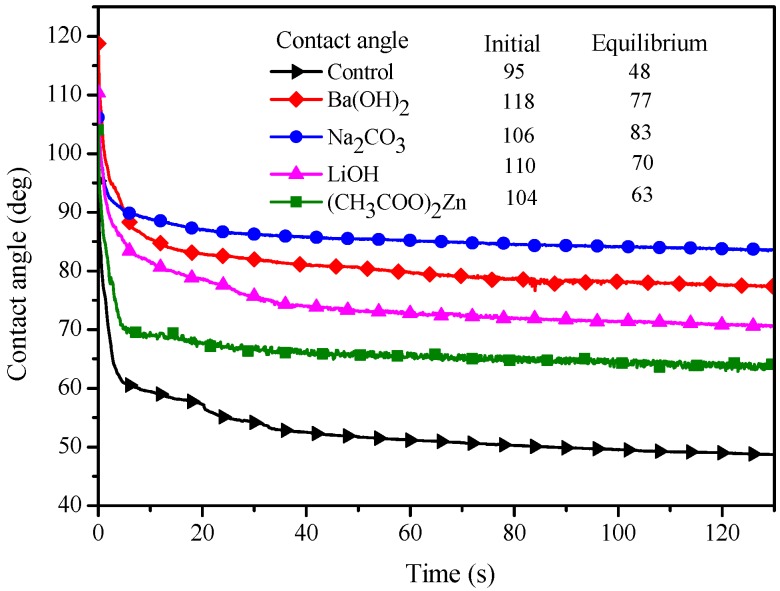
Contact angle as a function of time for the PF resins accelerated by different catalysts.

**Figure 2:**
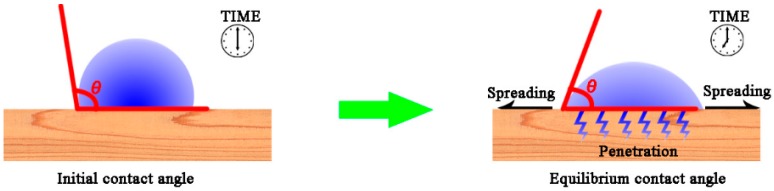
Manifestation of adhesive wetting process: contact, spreading, and penetration.

The wettability of PF resin on solid surface is usually evaluated by contact angle [13], which was tested on smoothed wood surface in this study. Due to liquid penetration and spreading on the wood surface, the contact angle changed as a function of time, as shown in Figure 1. The process of adhesive wetting includes three steps [14]: (1) formation of a contact angle at the solid and adhesive interface; (2) spreading of the adhesive over a solid surface; and (3) adhesive penetration into the porous solid substrate, as shown in Figure 2.

As shown in Figure 1, at the initial stage of the wetting process, the contact angle of the resins decreased quickly. As time elapsed, the contact angle decreased more slowly and finally attained relative equilibrium. It was observed that the Na_2_CO_3_-accelerated PF resin showed the largest equilibrium contact angle, and the resins could be ranked as Na_2_CO_3_ > Ba(OH)_2_ > LiOH > (CH_3_COO)_2_Zn > Control. The results suggested that the Na_2_CO_3_-accelerated PF resin had the largest surface tension, which may be due to its larger viscosity. Figure 1 also shows that the (CH_3_COO)_2_Zn-accelerated resin had the fastest rate of contact angle change, which meant that (CH_3_COO)_2_Zn-accelerated resin could spread and penetrate more quickly into the porous structure of wood. Both viscosity and the chemical constitution of PF resin could alter the contact angle change rate. Usually, samples with lower viscosity exhibit a faster contact angle change rate. However, from the data in Table 1, (CH_3_COO)_2_Zn-accelerated resin showed higher viscosity than that of the control sample, but had a faster contact angle change rate. Thus, the chemical constitution of (CH_3_COO)_2_Zn-accelerated PF resin may be the main factor altering its contact angle change rate. The hydroxymethyl of PF resin is the main chemical group that can easily connect with the hydroxyl of wood cellulose; thus, our data suggests that (CH_3_COO)_2_Zn-accelerated PF resin may contain more hydroxymethyl.

### 3.3. FT-IR Spectroscopy

**Figure 3:**
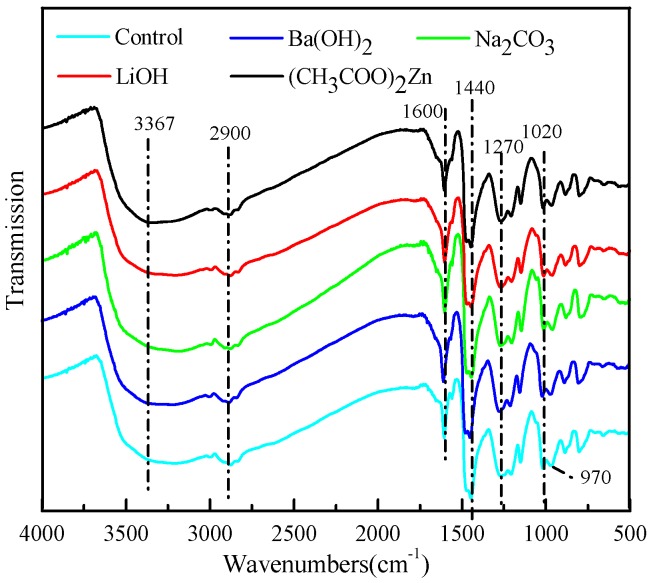
FT-IR spectra of the PF resins.

To investigate the structural changes in the PF resins accelerated by different catalysts, FT-IR spectra (Figure 3) were obtained after vacuum-drying the samples. The spectra assignments of the PF resins are shown in Table 2 [15,16,17].

There were no significant differences between the spectra of the catalyst-accelerated resins and the control sample, which indicated structural similarity. Bands at 1020 cm^−1^ were ascribed to C–O stretching vibration of aliphatic C–OH, aliphatic C–O, and methylol C–OH. Bands at 1600 cm^−1^ were assigned to the elongation of aromatic –C=C–, which were consistent in each reaction system and unaffected by catalyst reaction. Thus, bands at 1600 cm^−1^ could be used as an internal standard for analysis. The ratio of absorption value of 1020 cm^−1^ (variable)/1600 cm^−1^ (constant) was calculated to indicate the degree of hydroxymethyl for phenol in each catalyst accelerated-reaction system, as shown in Table 3. The control of PF resins had a relatively larger ratio of 1020 cm^−1^/1600 cm^−1^, which may be explained by the fact that the methylol of catalyst-accelerated PF resin tends to undergo further condensation reactions to form methylene (–CH_2_–). Thus, the control sample had relatively more unreacted methylol. This explanation was confirmed by the fact that the control sample showed the lowest viscosity due to its relatively minimum condensation degree.

### 3.4. Chemical Structure Analysis

**Figure 4:**
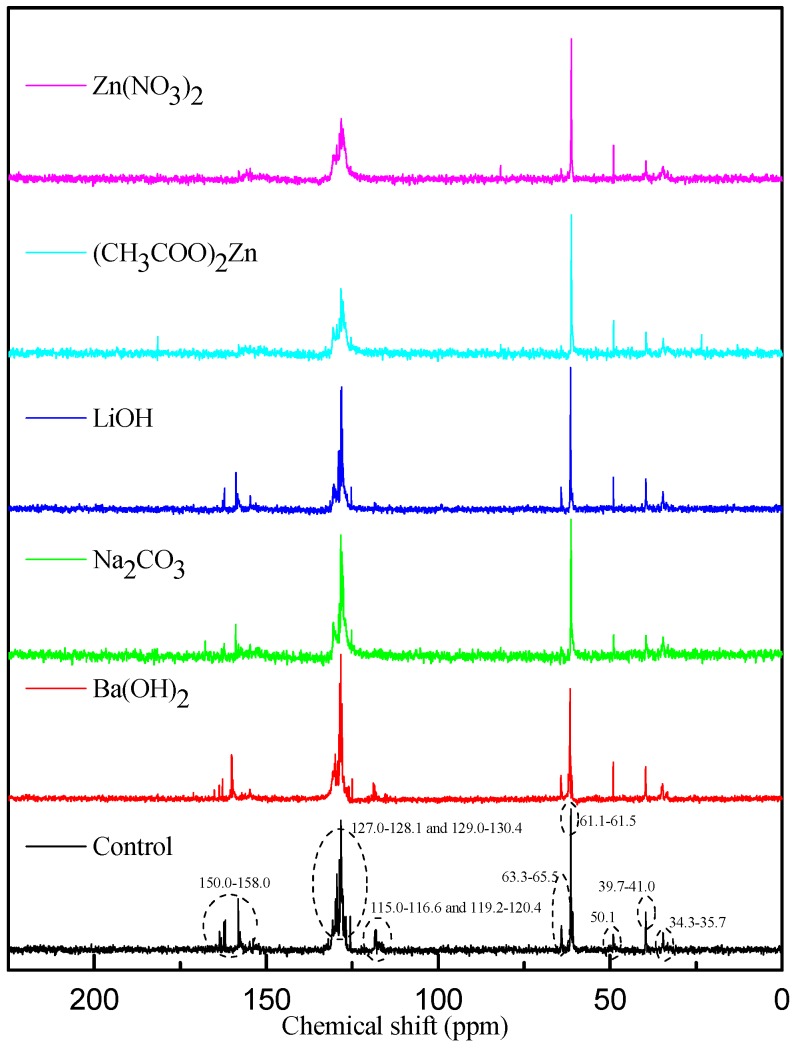
Liquid ^13^C NMR spectra of PF resins.

**Figure 5:**
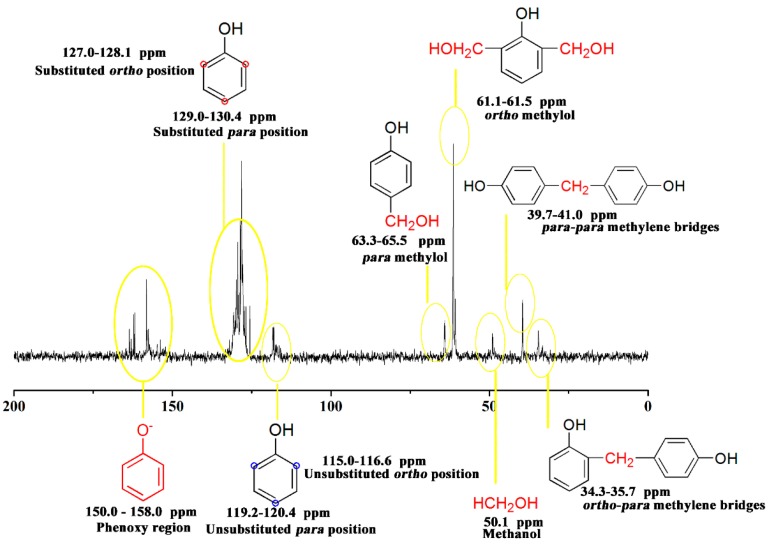
Liquid ^13^C NMR chemical shifts and group assignments of PF resin.

In order to identify the effect of different catalysts on the functional groups, quantitative ^13^C NMR was used to study the difference of chemical shifts between the control and catalyst-accelerated PF resins. The ^13^C NMR spectra are shown in Figure 4, and their corresponding assignments of groups’ signals are shown in Figure 5 [12,18,19,20].

The chemical shift of 150.0–158.0 ppm was assigned to phenoxy carbons (C1–OH), which was used as an integral standard and analytical standpoint. 156.2–156.8 ppm and 153.4–156.1 ppm were assigned to *para* alkylated groups and *ortho* alkylated groups, respectively. Usually, phenolic *ortho* and *para* carbons’ chemical shifts vary with the sodium hydroxide contents of the resin due to the ionization of phenoxy group and the kind of substituted groups. Substitution with methylol groups in the *para* and *ortho* carbon positions was shown at 129.0–130.4 ppm and 127.0–128.1 ppm. Unsubstituted *para* and *ortho* carbons, the main reactive sites for the methylolation reaction, occurred at 119.2–120.4 ppm and 115.0–116.6 ppm, respectively. The unsubstituted *para* and *ortho* carbon peaks were only present in the control and Ba(OH)_2_-accelerated PF resins, which indicated that Na_2_CO_3_, LiOH, and (CH_3_COO)_2_Zn facilitate the reaction of formaldehyde with phenolic *ortho* and *para* position more than what occurs in the control and Ba(OH)_2_ samples. A sharp peak of methanol was evident around 50 ppm for all resins. Industrial formaldehyde usually contains a small amount of methanol which can also be formed during resin synthesis from the Canizzaro reaction of formaldehyde. The signal peak of methylol is sharper than the methylene peak due to its higher group mobility and less variation in the environment within the polymer structure. Thus, the two peaks at 63.3–65.5 ppm and 61.1–61.5 ppm were assigned to *para* methylol and *ortho* methylol. Theoretically, condensation between two methylols can occur to form methylene ether bridges. However, the data in Figure 4 shows no peak between 69 and 74 ppm, indicating that methylene ether bridges were not formed between phenolic units during the synthesis of PF resin. The methylene bridges were easily observed in the range of 34–41 ppm. In a different chemical environment, different methylene linkages showed a different chemical shift, 39.7–41.0 ppm and 34.3–35.7 ppm were assigned to *para*–*para* and *ortho*–*para* methylene bridges, respectively. In order to remove the interference of carbon in CH_3_COO^−^ for ^13^C NMR analysis, the Zn(NO_3_)_2_-accelerated resin was also tested by ^13^C NMR analysis and used as a control for the ^13^C NMR analysis of (CH_3_COO)_2_Zn-accelerated resin.

Further analysis of quantitative ^13^C NMR is needed to elucidate the details of the cure-acceleration effect of different catalysts on the structure and compositions of PF resin. In this study, the ratios of integral values of the substituted position *ortho* (127.0–128.1 ppm)/*para* (129.0–130.4 ppm), *ortho* methylol (61.1–61.5 ppm)/*para* methylol (63.3–65.5 ppm), and methylene bridges *ortho-para* (34.3–35.7 ppm)/*para–para* (39.7–41.0 ppm) were calculated, as shown in Table 4. PF resins supplemented with Na_2_CO_3_, LiOH, and especially (CH_3_COO)_2_Zn, possessed higher ratios of *ortho*/*para*-substituted positions than did the control or the PF resin with Ba(OH)_2_. In case of the *ortho*/*para* ratio of methylol, the values of Na_2_CO_3_-accelerated and (CH_3_COO)_2_Zn-accelerated PF resins were not calculated (NC), because their ^13^C NMR spectra showed no signal for *para* methylol. Either Na_2_CO_3_ and (CH_3_COO)_2_Zn promoted the complete *para* methylol condensation reaction or drive formaldehyde toward the phenol *ortho* position exclusively. The ratio of *ortho* methylol/*para* methylol for the Zn(NO_3_)_2_-accelerated resin was much higher than other samples, excluding the Na_2_CO_3_-accelerated and (CH_3_COO)_2_Zn-accelerated PF resins. Usually, *para* methylol groups react more easily with other *para* positions to form *para–para* linkages. However, the data in Table 5 indicates that *ortho-para* linkages were equal to *para–para* linkages in the Na_2_CO_3_-accelerated, Zn(NO_3_)_2_-accelerated, and (CH_3_COO)_2_Zn-accelerated PF resins, suggesting that Na_2_CO_3_, Zn(NO_3_)_2_, and (CH_3_COO)_2_Zn were able to promote the condensation reaction to form *ortho-para* linkages. These results could be further proved by Table 1 that Na_2_CO_3_ and Zn(NO_3_)_2_-accelerated PF resins had higher viscosity, indicating that the significant promotion of phenol *ortho* position reactivity made Na_2_CO_3_ and Zn(NO_3_)_2_-accelerated PF resins have a tighter final network.

In conclusion, all the catalysts tested showed accelerating effect to promote phenol *ortho* reactivity. However, (CH_3_COO)_2_Zn and Na_2_CO_3_ were able to significantly promote the reaction activity of phenol *ortho* position and the condensation reaction of *ortho* methylol or directed formaldehyde exclusively toward the phenol *ortho* position.

### 3.5. Plywood Performance

**Figure 6:**
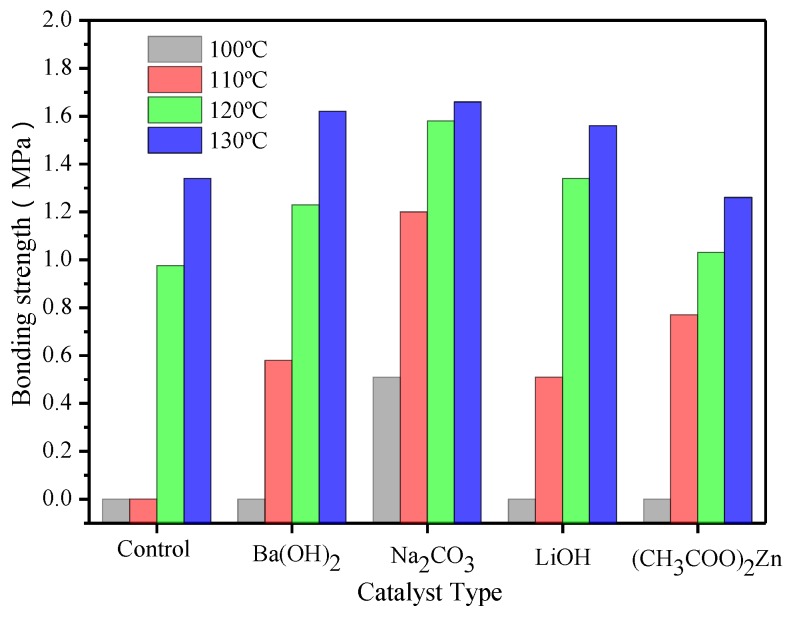
Effect of catalysts at different temperatures on the bonding strength.

Figure 6 shows the bonding strength of the plywood prepared with these different PF resins. Each kind of plywood was prepared at four hot-pressing temperatures, namely 100, 110, 120, and 130 °C. Higher hot-pressed temperatures allowed the resin to cure more completely and, thus, increase the bonding strength. Under the same hot-pressing temperature, the plywood prepared with catalyst-accelerated PF resins exhibited higher bonding strength than the control sample, especially the one with Na_2_CO_3_-accelerated PF resin. The data in Figure 6 shows that the plywood prepared with Na_2_CO_3_-accelerated resin at 110 °C yielded almost the same bonding strength of plywood with Ba(OH)_2_-accelerated resin at 120 °C, which is higher than that of the control sample pressed at 120 °C. The reason may be that the Na_2_CO_3_-accelerated PF resin had the highest viscosity among PF resins from Table 1, or that Na_2_CO_3_ significantly improved PF resin performance by promoting the reaction activity of phenol *ortho* position.

### 3.6. Thermal Behavior of the Cured PF Resins

**Figure 7:**
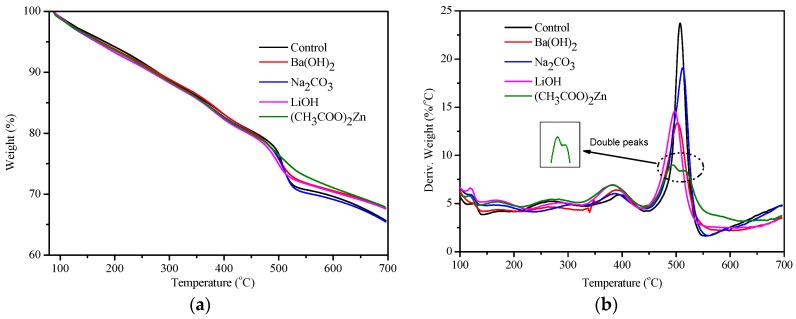
TG (**a**) and DTG (**b**) curves of the cured PF resins.

To characterize the thermal stability of the catalyst-accelerated PF resins, TG analysis was performed next, as shown in Figure 7. The temperatures at which the maximum degradation speed took place (*T*_max_) for the different thermal events of cured catalyst-accelerated PF resins are shown in Table 5.

It was previously known that phenolic resin degrades in three steps: post-curing, thermal reforming, and ring stripping [21,22]. The mass loss (about 5%) of the first thermal event at the lower temperature range (<155 °C) contributed to the evaporation of free water. In the second stage, with a temperature range from 230 to 300 °C, mass loss was due to the evaporation of water formed by the condensation reaction of methylol groups. The mass loss in the third event (from 350 to 440 °C) was due to the loss of water formed by the condensation reaction of methylol and phenolic hydrogen, as well as between two hydroxyl functional groups, which could cause further structure change of the cured products to a more tightly cross-linked network. In the fourth event (>450 °C), the mass loss was due to the loss of carbon monoxide and methane formed by degradation of the methylene linkage. As the temperature further increased, the remaining mass was from 65% to 68% at 700 °C, and that of Ba(OH)_2_, Na_2_CO_3_, and (CH_3_COO)_2_Zn-accelerated PF resins was higher than other samples, indicating that a tighter network and higher thermal stability was possessed by their molecular structure.

**Scheme 1:**
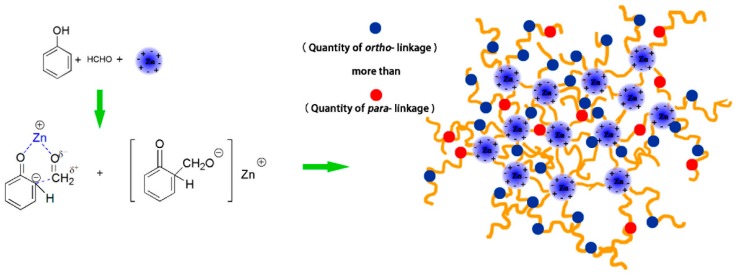
Possible synthesis mechanism and metal-ligand coordination morphology of the (CH_3_COO)_2_Zn/PF resin complex.

Figure 7 shows TG (a) and DTG (b) curves of the catalyst-accelerated PF resins. All of the PF resins showed similar thermal stability in the first three stages of thermal events. However, in the final event, the DTG curve of the (CH_3_COO)_2_Zn-accelerated resin showed lower degradation speed than the other PF resins, and had double peaks of degradation speed. According to Pizzi and Mohamed *et al.* [23,24,25], when a benzene ring was blended with a zinc ion, a complex compound between the phenolic nuclei and the zinc ion could be formed by a metal-ligand mode, which can accelerate the initial reaction of formaldehyde toward the phenolic nuclei by forming a carbocation of strong positive charge. As shown in Scheme 1, the mobility of the polymer chain was restricted by ion-polymer and ion-interaction, resulting in a higher thermal stability of PF resin than the control sample. Thus, the first of double peaks at 493 °C may indicate the breakage of the metal-ligand bonding mode, and the second peak of the double peaks at 518 °C may indicate the degradation of the methylene linkage.

## 4. Conclusions

The poly-condensations of PF resins with different catalysts suggested different abilities to accelerate the reaction. In general, the accelerating efficiency of the catalysts was Na_2_CO_3_ > (CH_3_COO)_2_Zn > Ba(OH)_2_ > LiOH.

The addition of Na_2_CO_3_ had a remarkable influence on the performance of PF resin. The viscosity of Na_2_CO_3_-accelerated PF resin increased to around 153 mPa·s quickly, five-fold greater than the viscosity of the control resin. Moreover, the gel time of PF resin decreased significantly and the bonding strength of plywood increased by the addition of Na_2_CO_3_. The quantitative ^13^C NMR analysis showed that the (CH_3_COO)_2_Zn and Na_2_CO_3_ catalysts could significantly promote the reaction activity of the phenol *ortho* position, and favor the condensation reaction of *ortho* methylol or direct formaldehyde toward the phenol *ortho* position exclusively. Compared with Na_2_CO_3_, the catalyst (CH_3_COO)_2_Zn showed a slightly weaker accelerating effect, but the contact angle analysis found that the (CH_3_COO)_2_Zn-accelerated resin showed a faster contact angle change rate, which represents a better wettability on the wood surface. Furthermore, the different peaks in the DTG curve and higher weight residue of TG data indicated that (CH_3_COO)_2_Zn has a different accelerating mechanism to improve the thermal stability of PF resin. That mechanism may include metal-ligand bonding between the benzene ring and zinc ion formed by ion-polymer and ion-interaction.

In conclusion, catalysts such as Na_2_CO_3_ and (CH_3_COO)_2_Zn showed significant accelerating effects to promote the curing of PF resin at lower temperatures and to improve PF resin performance. Thus, good catalyst-accelerated PF resins have promised to overcome the shortcoming of high curing temperature and to broaden their application.

## Figures and Tables

**Table 1:** PF resin characteristics.

Catalyst type	Performance		
	Solid content (%)	Viscosity (mPa·s)	Gel time (min)
Control	43	25.70	20.46
Ba(OH)_2_	46	73.70	15.57
Na_2_CO_3_	44	153.00	11.83
LiOH	46	58.30	15.88
(CH_3_COO)_2_Zn	44	81.00	13.98

**Table 2:** Assignments of FT-IR spectra of the PF resin.

Wavenumbers (cm^−1^)	Assignment
3,367	–OH stretching vibration
2,900	C–H stretching vibration of methylene
1,600, 1440	The elongation of aromatic –C=C–
1,270	C–O stretching vibration of phenolic C–OH and phenolic C–O
1,020	C–O stretching vibration of aliphatic C–OH, aliphatic C–O, and methylol C–OH
970	C–H stretching vibration of vinyl

**Table 3:** The ratio of absorption value of 1020 cm^−1^ (variable)/1600 cm^−1^ (constant) of the PF resins with different catalysts.

Wavenumbers (cm^−1^)	Absorption				
	Control	Ba(OH)_2_	Na_2_CO_3_	LiOH	(CH_3_COO)_2_Zn
1,020	43.46	32.63	33.79	45.43	29.85
1,600	29.75	28.14	29.94	38.54	24.81
Ratio (1,020/1,600)	1.46	1.16	1.13	1.17	1.20

**Table 4:** Liquid ^13^C NMR analysis results of PF resin formed with different catalysts.

PF resin	*ortho*/*para* (Substituted position)	*ortho*/*para* (Methylol)	*ortho-para*/*para–para* (Methylene bridges)
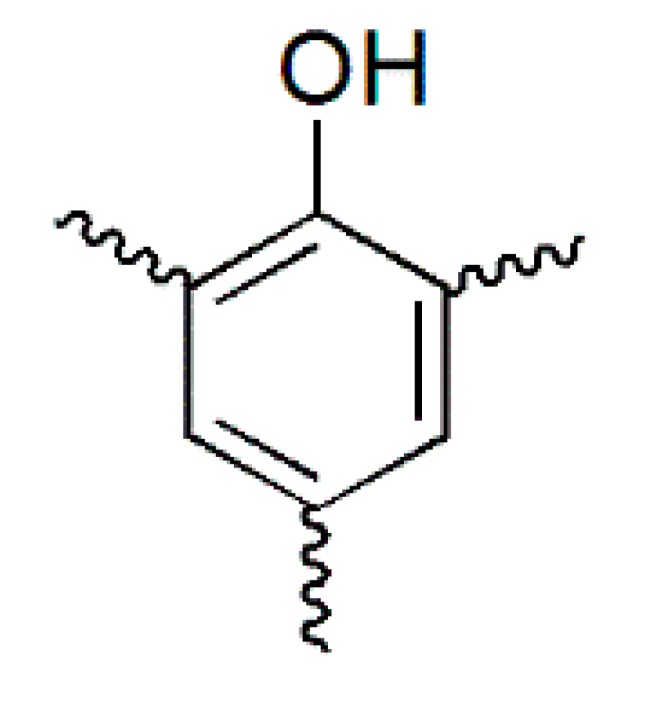	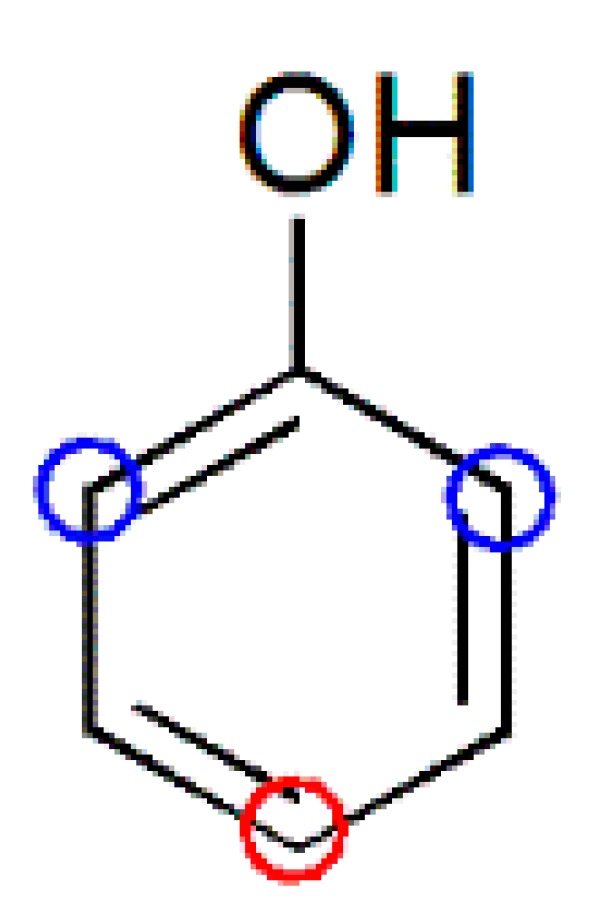	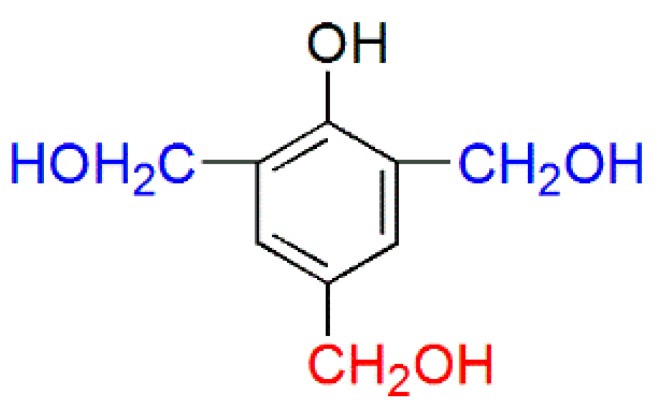	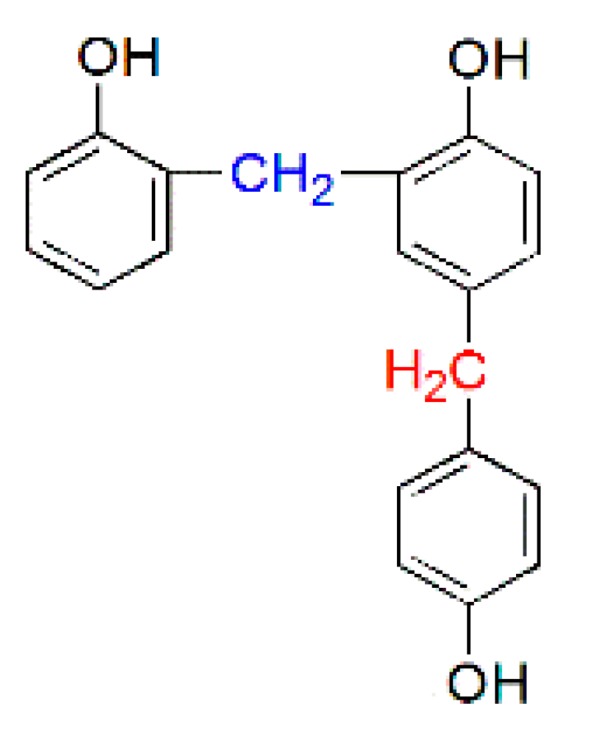
Control	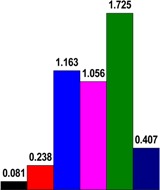	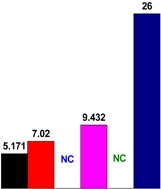	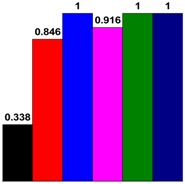
Ba(OH)_2_			
Na_2_CO_3_			
LiOH			
(CH_3_COO)_2_Zn			
Zn(NO_3_)_2_			

**Table 5:** Thermal properties of the cured PF resins.

Catalyst type	*T_max_* of Thermal event (°C)					Weight residue (%) at 700 °C
	First	Second	Third	Fourth	Fifth	
Control	155	260	394	507	–	65.5
Ba(OH)_2_	156	262	390	503	–	68.0
Na_2_CO_3_	153	300	386	512	–	65.5
LiOH	158	283	381	497	–	68.0
(CH_3_COO)_2_Zn	155	273	381	493	518	68.0
